# Downregulation of Cell Surface Major Histocompatibility Complex Class I Expression Is Mediated by the Left-End Transcription Unit of Fowl Adenovirus 9

**DOI:** 10.3390/v13112211

**Published:** 2021-11-03

**Authors:** Bryan D. Griffin, Juan Carlos Corredor, Yanlong Pei, Éva Nagy

**Affiliations:** Department of Pathobiology, Ontario Veterinary College, University of Guelph, Guelph, ON N1G 2W1, Canada; bryan.griffin@sri.utoronto.ca (B.D.G.); juan.corredor@sri.utoronto.ca (J.C.C.); ypei@ovc.uoguelph.ca (Y.P.)

**Keywords:** major histocompatibility complex class I, fowl adenovirus 9, left end transcription unit, inclusion body hepatitis

## Abstract

Major histocompatibility complex class I (MHC-I) molecules play a critical role in the host’s antiviral response by presenting virus-derived antigenic peptides to cytotoxic T lymphocytes (CTLs), enabling the clearance of virus-infected cells. Human adenoviruses evade CTL-mediated cell lysis, in part, by interfering directly with the MHC-I antigen presentation pathway through the expression of E3-19K, which binds both MHC-I and the transporter associated with antigen processing protein and sequestering MHC-I within the endoplasmic reticulum. Fowl adenoviruses have no homologues of E3-19K. Here, we show that representative virus isolates of the species *Fowl aviadenovirus C*, *Fowl aviadenovirus D*, and *Fowl aviadenovirus E* downregulate the cell surface expression of MHC-I in chicken hepatoma cells, resulting in 71%, 11%, and 14% of the baseline expression level, respectively, at 12 h post-infection. Furthermore, this work reports that FAdV-9 downregulates cell surface MHC-I through a minimum of two separate mechanisms—a lysosomal-independent mechanism that requires the presence of the fowl adenovirus early 1 (FE1) transcription unit located within the left terminal genomic region between nts 1 and 6131 and a lysosomal-dependent mechanism that does not require the presence of FE1. These results establish a new functional role for the FE1 transcription unit in immune evasion. These studies provide important new information about the immune evasion of FAdVs and will enhance our understanding of the pathogenesis of inclusion body hepatitis and advance the progress made in next-generation FAdV-based vectors.

## 1. Introduction

Viruses have evolved diverse strategies to counter the host’s antiviral defenses by targeting components of both the innate and adaptive immune responses [1]. While human adenovirus (HAdV) early genes such as E1A, E1B, E3, and E4 are well known for their roles in counteracting the host’s antiviral defenses through a variety of mechanisms, homologs to these genes are lacking in fowl adenoviruses (FAdVs) that infect avian species [2,3]. The FAdV genes that are responsible for immune evasion in infected chickens remain largely unknown.

Fowl adenoviruses (FAdVs) are in the family *Adenoviridae* and genus *Aviadenovirus*, comprising 15 species [4,5]. Some FAdVs, notably FAdV-2, FAdV-8a and -8b, FAdV-11, and some strains of FAdV-4, are associated with economically important poultry diseases such as inclusion body hepatitis (IBH) [6] and hepatitis hydropericardium syndrome (HHS) [7]. Conversely, some FAdV strains, such as FAdV-9 strain A-2A, cause mild or no disease and are routinely isolated from healthy flocks [5].

The complete nucleotide sequence of the FAdV-9 DNA genome as well as the predicted open reading frames (ORFs) and transcriptional map were described previously [3,8,9]. The left-end region of the viral genome consists of at least two transcriptional units: the fowl adenovirus early region 1 (FE1) consists of six rightward-oriented ORFs (ORFs 0, 1, 1A, 1B, 1C and 2), whilst the leftward-oriented ORFs (ORFs 24, 14, 13 and 12) are likely to be transcribed from the FE2 promoter [8]. Previous studies have described FAdV-9 deletion mutants, including FAdV-9∆4, which lacks all of the ORFs within the FE1 unit and replicates at the wild-type level in vitro [10]. In infected chickens, relative to wild-type FAdV-9 (wtFAdV-9), FAdV-9∆4 replicates at a lower titer and elicits a lower antibody response [10,11]. Recently, we reported that the viral dUTPase (ORF1) upregulates the expression of type I interferons in vitro [12] and seems to play roles in the immune response in infected chickens [13]. The FE1 region may, therefore, encode a cluster of proteins that are responsible for modulation of the host’s antiviral response. 

Major histocompatibility complex class I (MHC-I) molecules are expressed on the surface of nucleated cells as a heterotrimer that consists of a type I transmembrane glycoprotein heavy chain (BF2) and a noncovalently associated light chain (β_2_m) and a host- or pathogen-derived peptide fragment [14,15]. MHC-I molecules play a critical role in the host antiviral response by displaying virus-derived antigenic peptides to cytotoxic T lymphocytes (CTLs), allowing the recognition and clearance of virus-infected cells. To evade host CTL responses, a myriad of viral proteins, including adenoviral proteins, have evolved to target the many stages of the MHC class I antigen presentation pathway [14,15]. For example, HAdV E1A and E3-19K inhibit MHC-I transcription and prevent MHC-I export from the endoplasmic reticulum (ER) to the cell surface, respectively [16,17,18,19], while the myxomavirus MV-LAP and M-T4 reduce levels of MHC-I by degradation in endosomes and prevention of MHC-I egress from the ER, respectively [20]. Here, we examine the modulation of MHC-I surface expression in cells infected with representative isolates from three FAdV species, FAdV-4 (*Fowl aviadenovirus C*), FAdV-9 (*Fowl aviadenovirus D*) and FAdV-8a (*Fowl aviadenovirus E*), and further explore the effects of the FE1 transcription unit of FAdV-9 on the regulation of MHC-I expression.

## 2. Materials and Methods

### 2.1. Cells and Viruses

Chicken hepatoma (CH-SAH) cells and Leghorn male hepatoma (LMH) cells (ATCC CRL-2117) were maintained as previously described [21]. The parental, wild-type virus, FAdV-9 strain A-2A (ATCC VR-833), and the generation of the deletion mutant FAdV-9∆4 (nts 491 to 2782), and the corresponding rescued virus, resFAdV-9∆4, were described previously [10,21]. All viruses were propagated in CH-SAH cells. When an advanced cytopathic effect (CPE) was observed, the cultures were subjected to three freeze–thaw cycles, and the resulting clarified virus supernatants were stored at −80 °C. Virus aliquots were titrated in triplicate by a plaque assay in CH-SAH cells, as described previously [21].

### 2.2. Plasmids

The infectious clones of FAdV-9, FAdV-9∆4, and resFAdV-9∆4 were reported previously [10]. To construct the eukaryotic MHC-I expression vector expressing the chicken MHC-I (B21), pFLAG-CMV-1-MHC-I-B21, RT-PCR was performed using a Superscript III first-strand synthesis kit (Invitrogen) and the reverse primer 5′-CAGATGGCGGGGTTGCTCC-3′, according to the manufacturer’s instructions. The resulting cDNA was then amplified by PCR using KOD DNA polymerase (Novogen, Madison, WI, USA) and primers 5′-CAGGAATTCAGAGCTCCATACCCTGCGGTAC-3′ and 5′-CAGGAATTCTCAGATGGCGGGGTTGC-3′ that each contained EcoRI sites (underlined) and began after the predicted signal peptide cleavage site located between aa 21 and 22 of the MHC-I polypeptide, as predicted by Signal-P 3.0. To generate pGEM-MHC-I-B21, the obtained PCR product was cloned into the pGEM-T Easy vector (Promega, Promega, WI, USA), according to the manufacturer’s instructions. After sequence verification, pGEM-MHC-I-B21 was digested with EcoRI and the resulting fragment was subcloned into pFLAG-CMV-1 downstream of the Met-preprotrypsin-FLAG sequence, resulting in pFLAG-CMV-1-MHC-I-B21. The β-actin standard construct [22] was kindly provided by Dr. Shayan Sharif.

### 2.3. Antibodies

A mouse monoclonal antibody to the chicken MHC-I/BF2 (F21/2) (Southern Biotech, Birmingham, AL, USA, 8345); rabbit polyclonal antibodies to the human CD71, the transferrin receptor (TfR), (H-300) (Santa Cruz Biotech, Santa Cruz, CA, USA); goat polyclonal antibodies to the human actin (I-19) (Santa Cruz Biotech); mouse monoclonal antibody (MAb) to the HA tag (HA-7) (Sigma-Aldrich, St. Louis, MO, USA); and anti-FLAG M2 MAb (Sigma-Aldrich, St. Louis, MO, USA, F1804) were used according to the manufacturers’ instructions. 

### 2.4. Quantitative RT-PCR

CH-SAH cells (6.3 × 10^6^) were mock-infected or infected with the various viruses at a multiplicity of infection (MOI) of 5. Total RNA was extracted with Trizol (Invitrogen, Carlsbad, CA, USA), according to the manufacturer’s instructions. The relative levels of MHC-I and β-actin mRNAs were assessed using two-step RT-PCR and the comparative threshold cycle (∆∆Ct) method [23]. Briefly, two micrograms of total RNA was treated with DNase I (Fermentas) and reverse transcribed using Superscript III (Invitrogen, Carlsbad, CA, USA) with 50 ng of random primers (Invitrogen, Carlsbad, CA, USA) in a 20 µL reaction volume, according to the manufacturer’s instructions. For each cDNA synthesis reaction, a reverse transcription (RT)-negative control was performed to ensure that no residual viral genomic DNA remained following the DNase I treatment. The cDNA product was diluted (1:10) and 2 µL of the diluted product was amplified in a 20 µL reaction mixture with 10 µL of Lightcycler 480 SYBER Green Master Mix (Roche, Basel, Switzerland) and 2.5 µM (each) forward and reverse gene-specific primers as previously described [22,24]. The cycling conditions, melting curve analyses, and efficiencies for MHC-I and β-actin were previously reported [22,24]. Reactions were performed with a Lightcycler 480 (Roche, Basel, Switzerland), and the results were analyzed using the Lightcycler 480 software v1.5.0 (Roche, Basel, Switzerland). 

### 2.5. Western Blotting

CH-SAH cells were mock-infected or infected with the various viruses at an MOI of 5. Prior to infection, cells were either left untreated; transfected with Lipofectamine 2000 (LF) (Invitrogen, Carlsbad, CA, USA) alone or with LF and 0.33 µg polyriboinosinic:polyribocytidylic acid (poly(I:C) (Sigma, St. Louis, MO, USA, P0913) 9 h prior to infection, according to the manufacturer’s instructions, except that the liposome-containing medium was replaced with complete medium (5% FBS) 3 h after transfection; treated with dimethyl sulfoxide (DMSO) solvent control at 4 h post-infection (h.p.i.) or 0.1 µM bafilomycin A1 (Sigma-Adrich, St. Louis, MO, USA) in DMSO at 4 h.p.i.; or treated with 10 µM MG-132 (Calbiochem, Darmstadt, Germany) in DMSO at 9 h.p.i. Cells were harvested at 12 h.p.i. and lysed in radioimmunoprecipitation assay (RIPA) buffer [50 mM Tris-HCl (pH 7.5), 150 mM NaCl, 1% Triton X-100, 1% sodium deoxycholate, 0.1% sodium dodecyl sulfate, 10 mM EDTA], containing a protease inhibitor cocktail (Sigma, St. Louis, MO, USA). The protein concentrations of the lysates were determined by the Bradford method [25]. Proteins were resolved by 12% sodium dodecyl sulfate-polyacrylamide gel electrophoresis and transferred to 0.45 µm Amersham Hybond-P PVDF membranes (GE Healthcare, North Richland Hills, TX, USA). Blots were blocked overnight at 4 °C and probed with mouse MAb against the chicken MHC-I/BF2 (F21/2) (1:4000) or goat polyclonal antibodies to the human actin (I-19) (1:200) then washed and probed with goat anti-mouse IgG (H + L) horseradish peroxidase-conjugate (1:10,000; Invitrogen) or peroxidase-conjugated Affinipure donkey Anti-Goat IgG (H + L) (1:20,000; Jackson Immunoresearch, West Grove, PA, USA). Protein bands were visualized with Western Lightning Plus ECL (Perkin Elmer, Waltham, MA, USA) Western blotting detection reagents on a ChemiDoc XRS + (Biorad, Hercules, CA, USA). The intensities of the bands in the Western blot were quantified by densitometric analysis using Image Studio v5.2.5 Lite (Li-Cor Bioscience, Inc., Lincoln, NE, USA).

### 2.6. Flow Cytometry

CH-SAH cells were mock-infected or infected with the various viruses at an MOI of 5. At different times post-infection cells were gently detached with a 20–30 min incubation at 37 °C in calcium- and magnesium-free Hanks’ balanced salt solution (HBSS) containing 0.05% trypsin and 0.027% EDTA-2H_2_O (pH 7.4), and clumps were gently disrupted with repeated pipetting. An equal volume of complete medium (DMEM/F12) containing 10% dialyzed FBS was added to inactivate the trypsin. Cells were then washed once in complete medium (DMEM/F12) then washed once in phosphate-buffered saline (PBS) containing 2% bovine serum albumin (BSA) (PBS 2% BSA), and finally resuspended in PBS-2% BSA. Cells (1 × 10^6^) were incubated for 1 h at 4 °C with the mouse MAb MHC I (F21/2) (1:40), rabbit polyclonal antibodies to the transferrin receptor (TfR) (1:80), or mouse monoclonal anti-FLAG M2 antibody (1:40). After three washes, the cells were incubated in fluorescein (FITC) affinipure F(ab’)2 fragment goat anti-mouse IgG (H + L) (1:50) for 30 min at 4 °C in the dark. Cells were then washed three times with PBS-2% BSA and resuspended in PBS-2% BSA or PBS-2% BSA with propidium iodide (PI). Flow cytometry was performed on a FACScan flow cytometer (BD Biosciences, San Jose, CA, USA). For each sample, a minimum of 20,000 events were collected and all data were corrected for nonspecific background binding obtained with isotype controls. For each treatment, unstained cells and cells stained with each antibody or PI alone were used to create a compensation matrix that was applied to the data to correct for the spectral overlap of FITC and PI. All data were subsequently analyzed and compensated with FlowJo 7.6.5 software (TreeStar Inc., Ashland, OR, USA). All statistical analyses were performed using Graphpad Prism (GraphPad Software, Inc., San Diego, CA, USA). 

### 2.7. Internalization and Restoration Assays

To determine the rate of internalization of cell surface MHC-I, CH-SAH cells were infected (MOI of 5) and incubated at 37 °C until 4 h.p.i., when 1 × 10^5^ cells in 96-well plates were stained at 0 °C with saturating concentrations of anti-MHC-I MAb F21/2 and then washed and incubated at 37 °C for 15 or 30 min. The cells were then stained and processed, as above, except that flow cytometry was performed on a BD Accuri flow cytometer (BD Biosciences, San Jose, CA, USA).

To determine the kinetics of cell surface MHC-I restoration, CH-SAH cells were infected (MOI of 5) and incubated at 37 °C until 4 h.p.i., when live cells were left untreated or treated with ice-cold citrate buffer (0.062 M Na_2_HPO_4_, 0.132 M citric acid, 0.5% BSA, pH 3) for 2 min, as described previously [26], to denature and render surface MHC-I undetectable with the anti-MHC-I MAb. The acid solution was then neutralized by washing three times with DMEM/F12 10% FBS. The cells were incubated at 37 °C with or without DMSO solvent control or 0.1 µM bafilomycin A1 for the times indicated (0 or 8 h re-expression following surface MHC-I denaturation) when they were harvested for flow cytometric analysis on a FACScan flow cytometer (BD Biosciences, San Jose, CA, USA), as described above.

### 2.8. Immunofluorescence Microscopy

CH-SAH cells were seeded onto glass coverslips in 6-well plates at a density of 1.5 × 10^6^ cells per well. Cells were mock-infected or infected with the various viruses at an MOI of 5. Since the MHC-I epitope recognized by the mouse MAb to the chicken MHC I/BF2 (F21/2) was denatured upon fixation with 3.7% paraformaldehyde for 10 min at room temperature (RT) (data not shown), staining for extracellular MHC-I was instead performed on live cells. Briefly, at the indicated times, post-infection cells were stained with anti-MHC-I primary antibody (F21/2) diluted in complete medium with 5% FBS (1:100) at 4 °C for 1 h. Cells were washed 3 times in complete medium with 5% FBS at 4 °C then stained with goat anti-mouse IgG (H + L) Dylight 594 (Jackson Immunoresearch, West Grove, PA, USA) diluted in complete medium with 5% FBS (1:100) at 4 °C for 30 min. Cells were washed 4 times with PBS supplemented with 0.01% (*w*/*v*) MgCl_2_-6H_2_O and 0.0133% (*w*/*v*) CaCl_2_-2H_2_O and fixed in 3.7% paraformaldehyde in PBS at RT for 10 min. 

To detect intracellular and extracellular MHC-I, CH-SAH cells were transfected with the MHC-I expression vector, pFLAG-CMV-1-MHC-I-B21, or cotransfected with pFLAG-CMV-1-MHC-I-B21 and EYFP-Golgi at 12 h prior to infection, using Lipofectamine 2000 according to the manufacturer’s instructions. Cells were mock-infected or infected with the various viruses at an MOI of 5. At 12 h.p.i., cells were fixed with 3.7% paraformaldehyde in PBS and permeabilized in 0.1% NP40-PBS. Cells were washed 3 times in PBS and blocked for 1 h at RT in blocking solution (5% goat serum, 1% BSA in PBS), and then stained against the FLAG epitope with either anti-FLAG M2 MAb produced in mice or anti-FLAG MAb produced in rabbits and the KDEL ER Marker 10C3 produced in mice diluted in blocking solution (1:100) at RT for 2 h. Cells were then washed 3 times in PBS and stained with either goat anti-mouse IgG (H + L) DyLight 594 or goat anti-mouse DyLight 594 and Alexa Fluor^®^ 488 donkey anti-rabbit diluted in blocking solution (1:100) for 1 h at RT in the dark. All coverslips were washed 3 times in PBS and mounted onto slides with Prolong gold antifade reagent with DAPI (Invitrogen, Carlsbad, CA, USA) and examined with a Leica DM 6000B microscope with a 63× glycerol immersion objective connected to a Leica CSLM SP5 system (Leica Microsystems, Wetzlar, Germany) with Leica LAS AF Imaging software (Leica Microsystems, Wetzlar, Germany). The images were further processed, and overlays were generated with Image J (National Institutes of Health) with additional plugins from the McMaster Biophotonics Facility (Canada).

## 3. Results 

### 3.1. FAdV-4, FAdV-8a, and FAdV-9 Downregulate Cell Surface MHC-I

**Figure 1:**
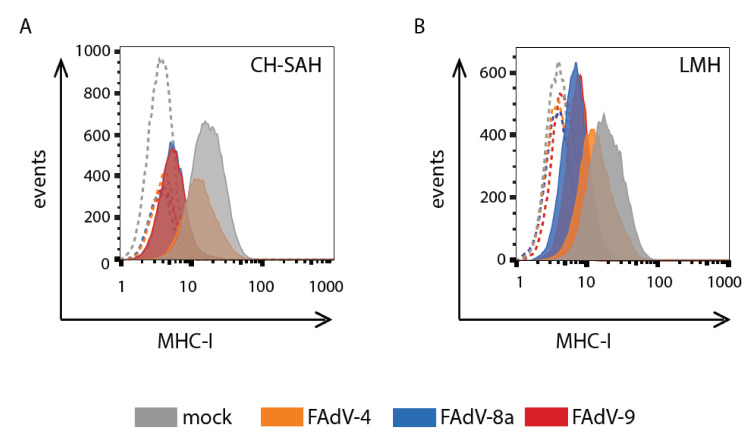
Surface MHC-I expression in FAdV-infected cells. Chicken hepatoma (CH-SAH) cells (**A**) and chicken Leghorn male hepatoma (LMH) cells (**B**) were either mock infected or infected with FAdV-4, FAdV-8a, or FAdV-9 at a multiplicity of infection of 5. Cell surface expression of MHC-I was analyzed at 12 h.p.i. using flow cytometry. Primary anti-MHC-I MAb F21/2 was used, followed by fluorescein isothiocyanate (FITC)-conjugated goat anti-mouse IgG. Live cells were gated on the basis of propidium iodide staining. The histrograms depict the number of cells detected at a particular fluorescence (FITC) intensity. Dashed histograms indicate background staining obtained with an isotype control FITC-conjugated antibody. Each panel depicts a representative experiment.

Since viral inhibition of the MHC-I antigen presentation pathway has been shown to be an important immune evasion strategy, especially for DNA viruses, we hypothesized that FAdVs would also downregulate cell surface MHC-I. We first evaluated the MHC-I cell surface levels in FAdV- and mock-infected CH-SAH and LMH chicken hepatoma cells by means of flow cytometry with an anti-MHC-I (F21/2) MAb. The mean fluorescence intensity (FITC) of unpermeabilized, mock-infected CH-SAH or LMH cells saturated with mouse anti-MHC-I MAb and FITC-conjugated anti-mouse antibodies was found to be greater than 20 fluorescence units, indicating a moderate level of cell surface MHC-I (Figure 1). The mean fluorescence intensity of anti-MHC-I-labeled CH-SAH cells at 12 h.p.i. with FAdV-8a or FAdV-9 (MOI of 5) was 2.9 and 2.3 fluorescence units, respectively, significantly lower than mock-infected cells at the same time post-infection (*p* < 0.01, chi-square) (Figure 1). Infection with FAdV-8a or FAdV-9 resulted in an 86% and 89% reduction in MHC-I on the cell surface, respectively, compared to the levels in mock-infected cells (set to 100%). CH-SAH cells infected with FAdV-4 (strain ON1) resulted in a lower (29%) reduction in mean cell surface MHC-I expression at 12 h.p.i. and only a 54% reduction at 24 h.p.i. (data not shown). Similar reductions in surface-expressed MHC-I, though to a lesser extent, were observed at 12 h.p.i. of LMH cells with FAdV-4, FAdV-8a and FAdV-9, resulting in a reduction in surface MHC-I of 39%, 67%, and 61%, respectively (Figure 1). Since FAdV-9 showed the greatest reduction in cell surface MHC-I following infection, our studies focused on this virus.

### 3.2. FAdV-9-Mediated Downregulation of Cell Surface MHC-I in CH-SAH Cells Depends in Part on the FE1 Transcription Unit

**Figure 2:**
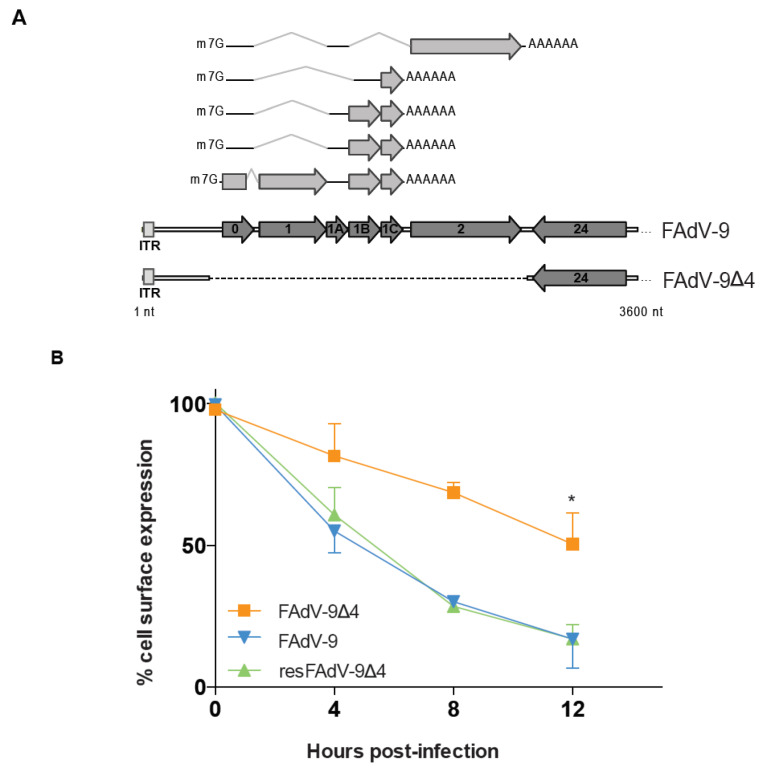

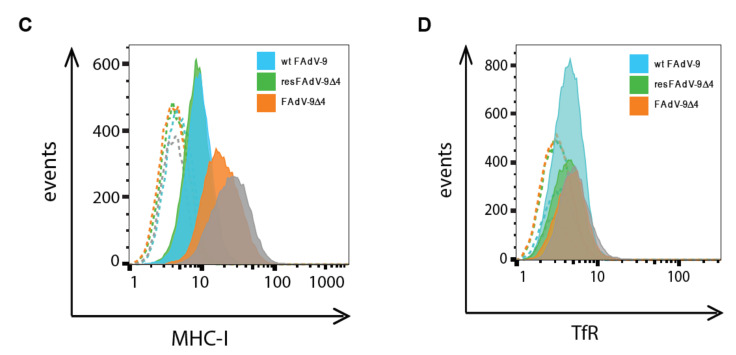
Role of the left-most transcription unit of FAdV-9 in regulating cell surface MHC-I and TfR. (**A**) The lower two ORF maps are schematic diagrams showing the left end genomic regions of FAdV-9 (and resFAdV-9∆4) and FAdV-9∆4. The dark grey arrows indicate the position and direction of transcription for the predicted ORFs, inverted terminal repeats are indicated by ITR. The transcripts resulting from alternative splicing, as previously reported [5] are shown above, to scale, with bold lines and light grey arrows representing exons and bent lines depicting introns. The 5′-m7G caps and polyadenylation signals are included for clarity. A dashed line indicates the genomic region that was deleted from FAdV-9∆4 (nts 491-2782), resulting in the complete removal of the leftmost transcription unit [7]. (**B**) Cell surface expression of MHC-I on CH-SAH cells was analyzed using flow cytometry at the times post infection indicated. The graphs present averages and standard deviations of the means obtained from three independent experiments and are shown as percentages of expression relative to mock-infected cells at each time point, which maintained initial levels of expression (100%). Background fluorescence was determined with an isotype control FITC-conjugated antibody for each treatment and subtracted. In some instances the errors were smaller than the symbols used in the graphs and are therefore not visible. An asterisk (*) indicates a significant difference (*p* < 0.05) by Student’s T test in comparison to the other two groups. (**C**) Cell surface expression of MHC-I on LMH cells was analyzed using flow cytometry at 12 h.p.i. after mock infection (grey) or infection with FAdV-9∆4 (orange), FAdV-9 (blue), and resFAdV-9∆4 (green) at an MOI of 5. Primary anti-MHC-I MAb F21/2 was used, followed by FITC-conjugated goat anti-mouse IgG. Live cells were gated on the basis of PI staining. Background fluorescence was determined with an isotype control FITC-conjugated antibody for each treatment (dashed lines). (**D**) Cell surface expression of TfR on CH-SAH cells was analyzed at 12 h.p.i. using flow cytometry after infection with FAdV-9∆4 (orange), FAdV-9 (blue), and resFAdV-9∆4 (green) at an MOI of 5. The dashed histograms depict the background staining obtained with isotype control FITC-conjugated antibody.

An FAdV-9 deletion mutant virus lacking the FE1 region that encodes ORF0, ORF1, ORF1A, ORF1B, ORF1C, and ORF2 (Figure 2A), named FAdV-9Δ4, replicates at wild-type levels in vitro [10]. However, relative to the wild-type virus, FAdV-9Δ4 replicates to lower titers in infected chickens based on decreased levels of viral genome copies in tissues and viral shedding in the feces [10]. Since downregulation of MHC-I molecules, at the transcriptional and post-translational levels, is required for efficient replication of human adenovirus in vivo [16,17,18,19], we hypothesized that insufficient virally induced downregulation of MHC-I expression and presentation of viral antigen at the cell surface contributed to the lower replication of FAdV-9Δ4 in infected chickens. To determine whether the FE1 transcription unit of FAdV-9 contributes to the downregulation of cell-surface MHC-I, CH-SAH cells were either mock-infected or infected with wtFAdV-9, FAdV-9∆4, and resFAdV-9∆4 followed by flow cytometric analysis (Figure 2B). At 4 h.p.i, MHC-I expression levels on the surface of cells infected with wtFAdV-9 or resFAdV-9∆4 were substantially reduced, specifically by 45% and 38% of the initial levels, respectively. In contrast, upon infection with FAdV-9∆4, the reduction in MHC-I expression was lower by only 18%. Between 4 and 8 h.p.i, cell-surface MHC-I was lost at levels of 6.2 ± 1.5, 8.1 ± 1.8 and 3.2 ± 2.2 h^−1^ fluorescence units in wtFAdV-9, resFAdV-9∆4 and FAdV-9∆4-infected cells, respectively. These fluorescence unit values represented reductions of 70%, 72% and 31% in cell-surface MHC-I in cells infected with wtFAdV-9, resFAdV-9∆4 and FAdV-9∆4, respectively. At 12 h.p.i., surface MHC-I levels were significantly reduced in all virus-infected cells compared to mock-infected cells (*p* < 0.01 Student’s T test). However, infection with wtFAdV-9 and resFAdV-9∆4 resulted in an 83% reduction, while a lesser decrease of only 50% was observed upon infection with FAdV-9∆4 (*p* < 0.01, Student’s T test). These results indicate that MHC-I antigen presentation is antagonized by FAdV-9, with at least one effector encoded within the FE1 transcription unit. Surface MHC-I was reduced similarly in LMH cells, when assessed at 12 h.p.i. (Figure 2C).

To ascertain the degree of specificity for FAdV-9-induced downregulation of MHC-I, we examined the expression of the cell surface presentation of the transferrin receptor (TfR), a membrane glycoprotein expressed at low levels that is nonetheless commonly used as a specificity control for MHC-I surface analyses [27]. At 12 h.p.i., the mean surface TfR levels were significantly reduced in all virus-infected cells compared to mock-infected cells (set to 100%, *p* < 0.01) (Figure 2D). However, while wtFAdV-9 and resFAdV-9∆4-infected cells showed a 54% and 45% reduction in mean fluorescence, respectively, FAdV-9∆4-infected cells exhibited a significantly lower (21%) reduction in TfR levels (*p* < 0.01, chi-square). These percent reductions reflect mean fluorescence values of 4.68 and 4.64 wtFAdV-9 and resFAdV-9∆4-infected cells, respectively, 5.19 for FAdV-9∆4-infected cells, and 5.7 for mock-infected cells before subtracting for background staining with isotype control secondary antibodies. Taken together, these results indicate that the mechanisms responsible for both FE1-dependent and FE1-independent surface MHC-I downregulation may not be restricted to just MHC-I.

### 3.3. FAdV Induced Downregulation of MHC-I Transcription Is FE1 Unit Independent 

**Figure 3:**
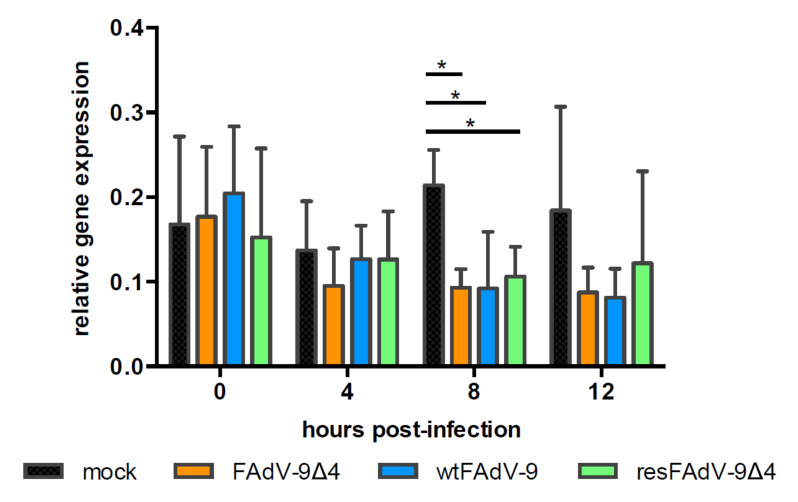
Quantitative RT-PCR of MHC-I (BF2) mRNA. CH-SAH cells were mock infected or infected with FAdV-9, FAdV-9∆4, and resFAdV-9∆4 at an MOI of 5. Total RNA was prepared from these cells at the times indicated post-infection and was subjected to quantitative real-time PCR with primers to MHC-I and β-actin. MHC-I mRNA levels were normalized to β-actin levels, and the resulting relative quantification is shown. Means ± SD from two independent experiments are shown. An asterisk (*) indicates a significant difference (*p* < 0.05) by Student’s T test in comparison to the other 3 groups at that time point.

To investigate whether the reduction in total MHC-I expression was due to the inhibition of transcription or the degradation of mRNA, the level of MHC-I heavy chain mRNAs relative to that of β-actin mRNAs was determined by a two-step quantitative RT-PCR (qPCR), as described previously [22,24]. MHC-I/BF2 transcript levels in virus-infected cells did not change at 0 and 4 h.p.i. relative to mock-infected cells (Figure 3). At 8 h.p.i, the all virus-infected cells had significantly reduced levels of MHC-I mRNA compared to mock-infected cells (0.21, 0.09, 0.09, and 0.11 for mock-, FAdV-9∆4, wtFAdV-9, and resFAdV-9∆4-infected cells, respectively). This suggests that virus infection reduces transcription of *mhc-1/bf2.* However, the overall reduction in MHC-I mRNA levels was similar in cells infected with FAdV-9∆4, which lacks FE1 or wtFAdV-9 and resFAdV-9∆4 that both contain FE1. This suggests that the reduction in MHC-I mRNA transcription in virus-infected cells is independent of FE1 ORFs.

### 3.4. FAdV Induced Downregulation of Total MHC-I Levels Is FE1 Unit Independent

**Figure 4:**
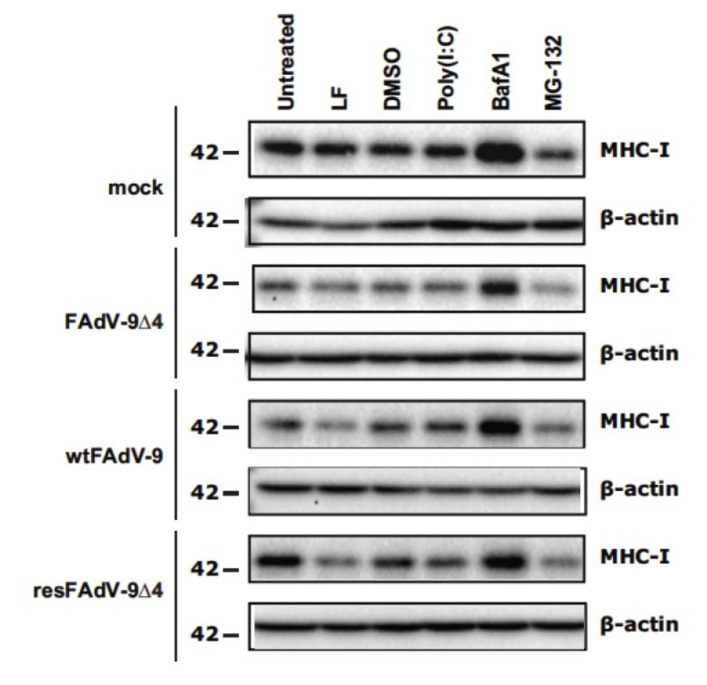
Western blot analysis of MHC-I heavy chain expression in virus-infected CH-SAH cells. Mock or FAdV-9, FAdV-9∆4, and resFAdV-9∆4-infected cells (MOI of 5) were either untreated, mock transfected with Lipofectamine 2000 (LF) (9 h prior to infection), treated with dimethyl sulfoxide (DMSO) solvent control (4 h.p.i.), transfected with polyriboinosinic: polyribocytidylic acid [poly(I:C)] (9 h prior to infection), treated with the lysosomal inhibitor bafilomycin A1 (BafA1) (4 h.p.i.), or treated with the proteosomal inhibitor MG-132 (9 h.p.i.). Whole cell lysates were harvested in RIPA buffer at 12 h.p.i. Cell lysates were analyzed by Western blotting for MHC-I expression. The levels of β-actin protein was determined and served as a loading control.

To evaluate the effect that FAdV-9 infection has on the levels of total MHC-I protein and the role of the FE1 region in this process, cell lysates of mock, FAdV-9∆4, wtFAdV-9, and resFAdV-9∆4-infected cells were analyzed by Western blotting (Figure 4). Mock-transfection with lipofectamine prior to infection caused a slight increase in total MHC-I in mock-infected cells and a decrease in all FAdV-infected cells (Figure 4, lane 2). Transfection of cells with polyinosinic-polycytidylic acid (poly I:C) with lipofectamine prior to infection, resulted in a similar amount of total MHC-I in mock-infected cells and an increased amount of total MHC-I in all virally infected cell lysates compared to lysates from lipofectamine-only-treated cells (Figure 4, lanes 4 and 2). At 12 h.p.i., the total MHC-I in lysates derived from FAdV-9∆4- and wtFAdV-9-infected cells showed a comparable reduction in the levels of total MHC-I expression, compared to lysates derived from mock-infected cells (Figure 4, lane 1), indicating that total MHC-I is reduced in FAdV-9-infected cells regardless of the presence or absence of the FE1 region. For unknown reasons, total MHC-I in resFAdV-9∆4-infected cells was not reduced to the same extent. Despite FAdV-9∆4 at 12 h.p.i. showing significantly more surface MHC-I than wtFAdV-9-infected cells (Figure 2), these data suggest that FE1 gene expression does not result in a net reduction in total MHC-I protein, but rather prevents MHC-I from reaching the cell surface, whereas an FE1-independent process results in a reduction in total MHC-I.

Mis-folded, mis-assembled, mis-localized, and non-recycled internalized MHC-I complexes are targeted for proteosomal or lysosomal degradation [28,29], and several viruses reroute MHC-I to these cellular compartments for degradation [14]. Therefore, the lysosomal inhibitor, BafA1, and the proteosomal inhibitor, MG-132, were employed to elucidate the involvement of these pathways for FE1-independent MHC-I degradation. As a control, cells were treated with DMSO, the solvent vehicle for both BafA1 and MG-132, which did not affect the levels of total MHC-I in mock- or virus-infected cells. Cells were infected at MOI of 5 and treatment with BafA1 or MG-132 started at 4 or 9 h.p.i., for a total of 8 or 3 h of exposure to the inhibitors, respectively. Lysates from uninfected and infected cells treated for 3 h with MG-132 showed a decrease in total MHC-I (Figure 4, lane 6). Lysates from all mock- and FAdV-9-infected cells treated with MG-132 showed a reduction in total MHC-I compared to those treated with DMSO. Since the mock-infected cells also showed a reduction in total MHC-I, we suggest that the inhibitor may be interfering with the loading of self and antigenic peptide onto MHC-I. Lysates from mock-infected cells that were treated with BafA1 had higher levels of total MHC-I than their untreated counterparts (Figure 4, lane 5). We therefore suggest that the inhibitor may be interfering with the normal turnover of MHC-I. Cell lysates from FAdV-9 and resFAdV-9∆4, and FAdV-9∆4-infected cells treated with BafA1 had increased MHC-I levels compared to DMSO-treated cells, suggesting that the degradation of MHC-I can be overcome with the inhibition of the lysosome.

### 3.5. The Presence of the FE1 Transcription Unit of FAdV-9 Does Not Affect the Rate of MHC-I Internalization, but Reduces the Rate of MHC-I Cell Surface Restoration

**Figure 5:**
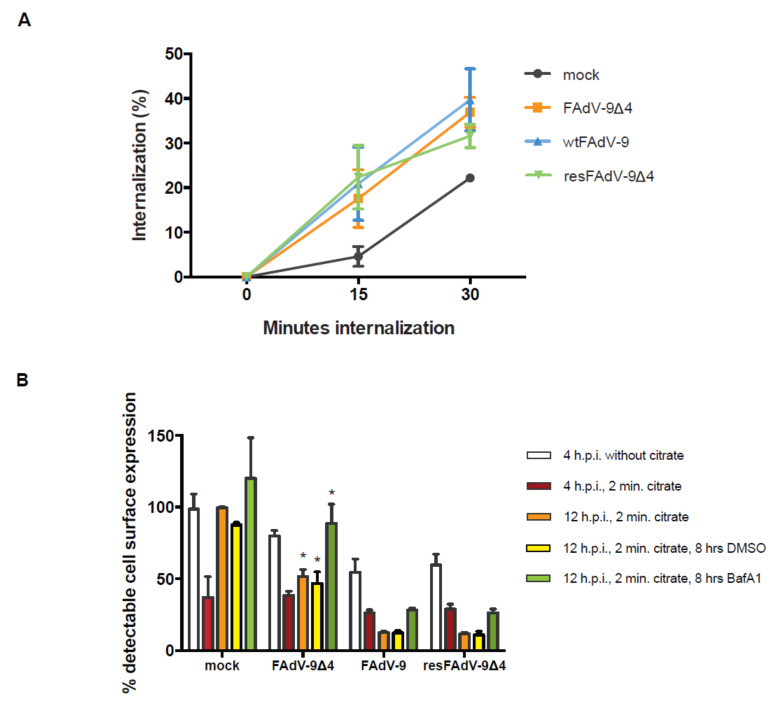
MHC-I internalization and the role of FE1 in appearance of MHC-I on the cell surface. (**A**) Assay for the extent of internalization of cell surface MHC-I. CH-SAH cells were either mock-infected or infected with FAdV-9∆4 (orange), FAdV-9 (blue), and resFAdV-9∆4 (green) at an MOI of 5. At 4 h.p.i., cells were incubated at 0 °C with saturating concentrations of anti-MHC-I MAb F21/2 and then washed and incubated at 37 °C for 15 or 30 min. The cells were then stained with FITC-conjugated goat anti-mouse IgG and analyzed by flow cytometry. Live cells were gated on the basis of propidium iodide staining. Background fluorescence was determined with an isotype control FITC-conjugated antibody for each treatment which was subtracted. The data points present averages and standard deviations of the means obtained from two independent experiments and are shown as percentage internalized relative to the initial MHC-I level at 4 h.p.i. (i.e. mean fluorescence following 30 min internalization for mock-infected cells minus mean fluorescence at 4 h.p.i for mock infected cells) (**B**) Restoration of cell-surface MHC-I following citric acid treatment. CH-SAH cells were either mock-infected or infected with FAdV-9∆4, FAdV-9, and resFAdV-9∆4 at an MOI of 5. Cells were then incubated at 37 °C until 4 h.p.i. when live cells were left untreated or treated with ice-cold citrate buffer for 2 min. The acid solution was then neutralized and the cells were incubated at 37 °C with or without DMSO solvent control or 0.1 µM Bafilomycin A1 for the times indicated (0.8 h re-expression). The cells were then stained with anti-MHC-I MAb F21/2 followed by FITC-conjugated goat anti-mouse IgG. Live cells were gated on the basis of PI staining. Background fluorescence was determined with an isotype control FITC-conjugated antibody for each treatment and subtracted. The bars present averages and standard deviations of the means obtained from two independent experiments and are shown as percentages relative to initial mock expression prior to citrate treatment. An asterisk (*) indicates a significant difference (*p* < 0.05) by Student’s T test in comparison to the corresponding treatment of the FAdV-9 and resFAdV-9∆4-infected cells.

We next performed studies to determine if FAdV-9 downregulates cell surface MHC-I by enhancing the rate of internalization and to evaluate the importance of the FE1 unit in this process. The rate of internalization was assessed by staining the cell surface of mock- and wild-type or knockout virus-infected cells at 4 h.p.i. with MAb against MHC-I (at 4 °C) followed by incubation for 0, 15, or 30 min at 37 °C followed by staining with the FITC-conjugated secondary antibody and flow cytometric analysis. MHC-I on the surface of cells infected with FAdV-9∆4, wtFAdV-9, and resFAdV-9∆4 was internalized at a similar rate (Figure 5A). While the rate of MHC-I internalization in virus-infected cells did not differ significantly from mock-infected cells, there was a trend towards an increased rate of internalization in infected compared to mock-infected cells. Since the rate of internalization was the same with or without FE1, the data suggest that one or more viral products encoded outside of the FE1 region may drive the acceleration of cell-surface MHC-I internalization.

Next, an experiment was carried out to determine the effect of the FE1 unit on the ability of de novo synthesized MHC-I to reach the cell surface [26]. At 4 h.p.i, mock-, FAdV-9∆4-, wtFAdV-9-, and resFAdV-9∆4-infected cells were incubated for 2 min in ice-cold citric acid buffer, which resulted in a significant reduction in detectable MHC-I on the cell surface, indicating denaturation of surface MHC-I (Figure 5B). Following the acid treatment, the cells were incubated at 37 °C in medium supplemented with PBS, DMSO, or BafA1 for 8 h until 12 h.p.i., at which point the cells were harvested for flow cytometric analysis. Cells were subsequently incubated with a MAb against MHC-I followed by staining with anti FITC-conjugated secondary antibody and were analyzed by flow cytometry. At 12 h.p.i., cell surface MHC-I on mock-infected cells treated with PBS, DMSO, or BafA1 had recovered to approximately 100% of the initial level at 4 h.p.i. prior to acid treatment through de novo synthesis. Interestingly, under the same conditions (PBS or DMSO treatment), FAdV-9∆4-infected cells showed a partial restoration of cell surface MHC-I, whereas wtFAdV-9/resFAdV-9∆4-infected cells showed a net loss of surface MHC-I, displaying less than 15% of the initial levels (the levels of surface MHC-I displayed at 4 h.p.i. prior to acid treatment) (Figure 5B). Cells infected with either wtFAdV-9 or resFAdV-9 that were treated with BafA1 did not reach the pre-acid treatment levels of cell surface MHC-I. Conversely, the FAdV-9∆4-infected cells treated with BafA1 recovered ~110% of the initial amount displayed at 4 h.p.i. prior to acid treatment. FAdV-9∆4-infected cells displayed 3-fold more surface MHC-I than the parental and rescued virus-infected cells at 12 h.p.i following the 8 h exposure to BafA1. These observations suggest that at least two separate mechanisms are responsible for surface MHC-I downregulation—an FE1-mediated process that is lysosome-independent, and an FE1-independent process that is lysosome-dependent.

### 3.6. The FE1 Transcription Unit of FAdV-9 during Infection Enhances Accumulation of MHC-I in the Endoplasmic Reticulum

**Figure 6:**
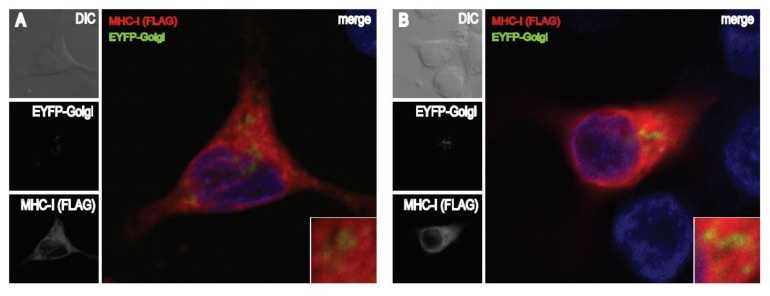

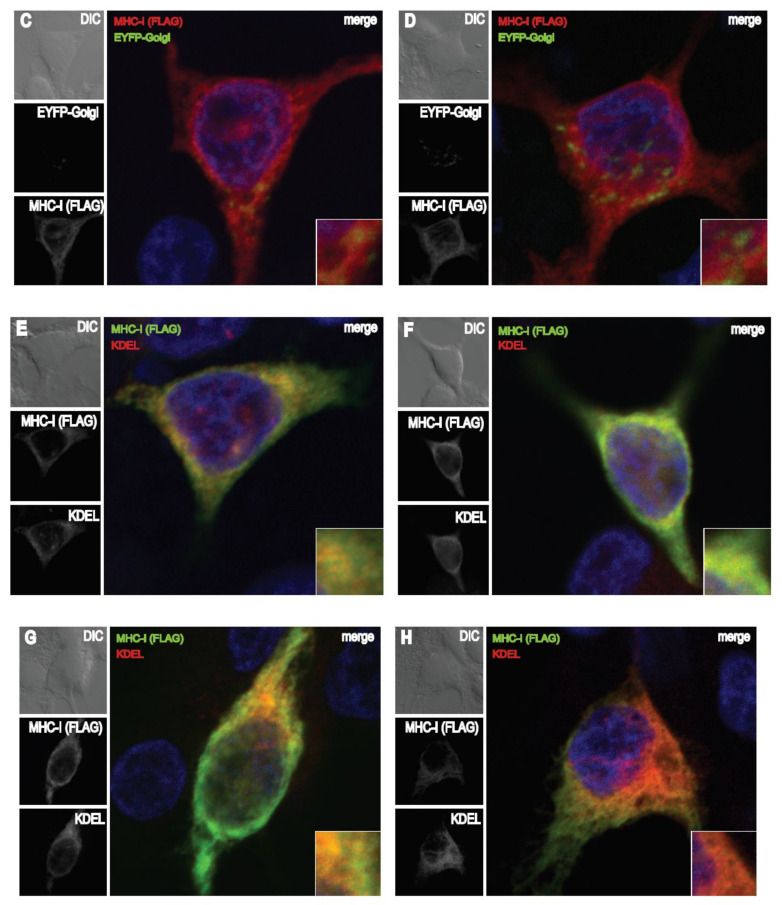
MHC-I localization in infected CH-SAH cells. Cells on coverslips were co-transfected with pFLAG-CMV-MHC-I-B21 and EYFP-Golgi (**A**–**D**) or with pFLAG-CMV-MHC-I-B21 (**E**–**H**) and 12 h later were mock infected ((**A**) or (**E**)) or infected with FAdV-9∆4 ((**B**) or (**F**)), wtFAdV-9 ((**C**) or (**G**)), and rescued viruses ((**D**) or (**H**)). At 12 h.p.i. cells were fixed, permeabilized, and stained for mouse MAb anti-FLAG M2 (**A**–**D**) or rabbit anti-FLAG and mouse KDEL ER Marker 10C3 (**E**–**H**) then stained with Dylight 594 goat anti-mouse (**A**–**D**) or Dylight 594 goat anti-mouse and Alexa Fluor^®^ 488 donkey anti-rabbit (**E**–**H**). All cells were mounted on slides using Prolong Gold Antifade with DAPI. The stained samples were analyzed and imaged by confocal laser scan microscopy using an inverted Leica SP5 microscope with a 63× glycerol immersion lens.

To follow how FE1 gene expression affects intracellular retention of MHC-I, the subcellular localization of FLAG-tagged MHC-I protein was determined by immunofluorescence microscopy. CH-SAH cells were either co-transfected with the MHC-I expression vector, pFLAG-CMV-1-MHC-I-B21, and the Golgi apparatus marker, EYFP-Golgi, (Figure 6A–D) or transfected with pFLAG-CMV-1-MHC-I-B21 alone (Figure 6E–H) and 12 h later were subsequently mock-infected (Figure 6A or Figure 6E) or infected with FAdV-9∆4 (Figure 6B or Figure 6F), wtFAdV-9 (Figure 6C or Figure 6G), or resFAdV-9∆4 (Figure 6D or Figure 6H). The cells were then stained to detect either FLAG-MHC-I alone with a FLAG-specific antibody (Figure 6A–D) or a FLAG-specific antibody and the KDEL ER marker 10C3 (Figure 6E–H). FLAG-MHC-I (red fluorescence in panels A to D) was distributed throughout the cytoplasm in mock-infected and parental or mutant FAdV-9-infected cells. Green fluorescent punctae, indicative of Golgi localization, was observed in panels A to D. No yellow or white punctate spots were identified, indicative that little FLAG-MHC-I was co-localized within the Golgi apparatus. FLAG-MHC-I (green fluorescence in panels E to H) and the KDEL marker (red fluorescence in panels E to H) were both distributed throughout the cytoplasm in mock-infected and parental or mutant FAdV-9-infected cells. As indicated by the yellow and white coloration seen in the merged image (Figure 6E–H, large panels), FLAG-MHC-I staining colocalized with KDEL staining at 12 h.p.i. (Figure 6E–H). These data indicate that a large proportion of intracellular FLAG-MHC-I protein was located within the ER. Since the colocalization of FLAG-MHC-I with KDEL staining was comparable in all the virally infected treatments, we could not determine whether or not the FE1 region directed sequestration of MHC-I within the ER; however, the data are consistent with this mechanism of action.

## 4. Discussion

To our knowledge, this is the first study demonstrating the ability of FAdVs to downregulate cell surface MHC-I during their replication cycle. Bioinformatic analyses of the right end of the FAdV-1 genome have suggested immunomodulatory functions for a cluster of ORFs (ORFs 9, 10 and 11) that are predicted to encode proteins with an immunoglobulin G-like structure [30], and this genomic region as a whole was shown to be important for virus replication in vivo [31]. However, to date, there have been no studies showing the immunomodulatory functions of these ORF products. The only ORF that has been published demonstrating an immunomodulatory function is FAdV-9 ORF1, a functional dUTPase that upregulates the expression of type I interferons in vitro [12].

Since the FAdV-9∆4 deletion mutant that lacks the entire FE1 transcription unit replicates to wildtype levels in chicken hepatoma cell lines but shows reduced replication in infected chickens [10], we hypothesized that the FE1 region encodes one or more gene products that interfere with MHC-I-mediated antigen presentation and are for this reason required to achieve wildtype replication in vivo. We, therefore, first sought to determine if FAdVs are capable of downregulating surface MHC-I and then subsequently investigated the role of the FE1 region in this process. 

This work demonstrated that MHC-I downregulation varies among the analyzed FAdV species. For example, cell surface MHC-I levels were significantly lower in cells infected with FAdV-9 and FAdV-8a (members of the species *Fowl aviadenovirus D* and *Fowl aviadenovirus E,* respectively) relative to those levels from cells infected with FAdV-4 (*Fowl aviadenovirus C*) (Figure 1). Furthermore, we observed that the replication cycle of FAdV-4 in CH-SAH cells was slower than that of FAdV-8a and FAdV-9 (data not shown). Our group has previously reported that FAdV-4 lacks an ORF1C homolog within its putative FE1 unit [32,33] that is present in both FAdV-8a and FAdV-9. We, therefore, speculate that the lower level of MHC-I downregulation in FAdV-4-infected cells could be due to relatively slower replication kinetics and/or the absence of ORF1C. ORF1C shares a similar structure to bovine papillomavirus (BPV) E5, which downregulates MHC-I [34] in addition to being a potent oncogene [35]. Therefore, these preliminary studies suggest a potential role for ORF1C in the downregulation of MHC-I surface expression. 

We first demonstrated that the level of cell surface MHC-I reduction was lower in FAdV-9Δ4-infected versus wt FAdV-9 or resFAdV-9Δ4-infected chicken hepatoma cells (Figure 2). FAdV-9∆4-infected cells at 12 h.p.i. displayed significantly more surface MHC-I than wtFAdV-9- or resFAdV-9Δ4-infected cells. While MHC-I transcription levels significantly decreased in all virus-infected cells at 8 h.p.i., deletion of the FE1 unit showed no difference in *mhc-I* transcription with respect to those of the wild type and rescued viruses (Figure 3). We therefore conclude that the reduction in *mhc-I* transcription is independent of FE1. Further, total MHC-I was reduced in parental or mutant FAdV-9-infected cells regardless of the presence or absence of the FE1 region (Figure 4). These data suggest that FE1 gene expression did not result in a net reduction in total MHC-I, but rather, prevented MHC-I from being expressed at the cell surface, whereas an FE1-independent process resulted in a reduction in total MHC-I that was caused at least in part by a reduction in *mhc-I* transcription. We found that the rate of MHC-I internalization appeared to increase in cells infected with parental or mutant FAdV-9, again regardless of the presence or absence of the FE1 region (Figure 5A); however, this observed increase was not statistically significant. An assay that measured de novo re-expression of surface MHC-I following chemical ablation revealed that in the presence of the lysosomal inhibitor, BafA1, CH-SAH cells infected with either wtFAdV-9 or resFAdV-9 failed to re-express surface MHC-I, while cells infected with FAdV-9∆4 were able to re-express surface MHC-I (Figure 5B). We therefore concluded that the expression of FE1 gene products appears to prevent newly synthesized or recycled MHC-I from reaching the plasma membrane. Immunofluorescence analysis of overexpressed MHC-I in CH-SAH cells with organelle markers specific for the Golgi apparatus or the ER showed that FE1-mediated intracellular sequestration of MHC-I does not occur in the Golgi apparatus but rather appears to be consistent with sequestration in the ER (Figure 6). This hypothesis could not be confirmed with certainty. However, peptide loading onto MHC-I occurs in the ER, and overexpressed FLAG-MHC-I was present in large amounts in the ER of both uninfected and parental or mutant virus-infected CH-SAH cells. The only commercial MAb available against the chicken MHC-I (F21/2) failed to detect MHC-I following formalin fixation of CH-SAH cells, which necessitated our using the eukaryotic expression vector, pFLAG-CMV-1-MHC-I-B21, to visualize FLAG-MHC-I.

As a first step, this work established an important role for the FE1 transcription unit in downregulating surface MHC-I expression, and future studies to determine the gene(s) responsible for FE1-mediated surface MHC-I downregulation would be warranted and interesting. We have recently shown that the viral dUTPase, encoded by ORF-1, upregulates the type I IFN response [12], known to upregulate the expression of MHC-I [36]. ORF-1-knockout mutant virus and FAdV-9Δ4 have similar effects in vivo in terms of decreased virus replication and antibody response [10,13]. Furthermore, as discussed above, ORF1C is a homolog of the BPV E5 oncoprotein that is responsible for activating the platelet-derived growth factor β receptor [37] and has also been shown to irreversibly retain MHC-I in the Golgi apparatus [38]. This is in contrast to FAdV-9 FE1-mediated intracellular MHC-I retention, which is both reversible and not within the Golgi apparatus (Figure 5 and Figure 6).

Proteins from several viruses target MHC-I for degradation in the lysosome, such as HIV Nef [39], human herpes virus 7 U21 [40], and Kaposi’s sarcoma-associated herpesvirus k3 and k5 [41]. The observation that de novo surface MHC-I was expressed in wtFAdV-9- or resFAdV-9-infected cells in the presence of the lysosomal inhibitor, BafA1, suggests that gene products encoded outside of FE1 may target MHC-I for degradation in the lysosome although it remains unclear to what extent the exposure of CH-SAH cells to BafA1 interferes with the normal turnover of MHC-I. Additional studies are currently underway to generate targeted deletions of predicted ORFs encoded outside of FE1 [42,43], and these mutant viruses will be screened for the loss of the ability to reduce total MHC-I during infection.

FAdV-9 appears to target MHC-I by two or more seemingly redundant strategies: FE1-dependent intracellular sequestration and FE1-independent reduction in total MHC-I. However, there is precedence for this with other DNA viruses, including HAdV, which encodes E1A and E3-19K, which inhibit MHC-I transcription and prevent the MHC-I export from the ER to the cell surface, respectively [16,17,18,19], and human cytomegalovirus, which interferes with MHC-I expression through four separate gene products [44]. FAdV-1 ORF22 and GAM-1 have functional similarity with mastadenovirus E1A [45], but a role in downregulating MHC-I has not been reported for either protein.

Lastly, since the surface expression of TfR, which is known to internalize through a clathrin-dependent endocytic process (in contrast to MHC-I that is internalized through a clathrin-independent endocytic process), was also reduced during FAdV infection, we suggest gene products encoded within FE1 may globally interfere with the secretory pathway. Other viruses similarly antagonize the secretory pathway, including norovirus [46], rotavirus [47], foot and mouth disease virus [48], coxsackievirus B3 [49], human rhinovirus 16 [50] and poliovirus [51]. 

The in vivo effects of the FE1 unit on MHC-I expression and its implication in replication and pathogenesis have not been studied. However, we have previously shown that chickens infected with FAdV-9Δ4 have lower anti-FAdV IgG antibody responses, virus genome copy numbers in tissues, and virus titers in the feces, compared to chickens infected with wtFAdV-9 and resFAdV-9Δ4 [10,13]. These observations could be attributed to the inability of FAdV-9Δ4 to efficiently downregulate MHC-I molecules due the absence of the FE1 unit. Consequently, FAdV-9Δ4-infected cells would be more prone to MHC-I-mediated antigen presentation, recognition, and clearance by CTLs. 

In conclusion, this is the first study to report the effects of FAdVs on the downregulation of surface expression of MHC-I molecules. Our data suggest that one of the mechanisms for reduced MHC-I during infection is an FE1-driven process that results in intracellular sequestration of MHC-I that could be attributed to a generalized block in the secretory pathway. This work provides the foundation for further studies on FAdV pathogenesis and the development of next-generation FAdV-based vaccines with increased efficacy.

## Data Availability

All relevant data are available from the authors upon request. For figures where raw data was not depicted source data is available at 10.6084/m9.figshare.16920454.
